# Rare primary pulmonary mucosa-associated lymphoid tissue lymphoma misdiagnosed with tuberculosis: A case report

**DOI:** 10.1097/MD.0000000000036125

**Published:** 2023-11-17

**Authors:** Min Gu, Dongze Ji, Yanfei Lu, Guoqiang Ping, Chengjing Yan

**Affiliations:** a Department of Laboratory Medicine, the First Affiliated Hospital of Nanjing Medical University, Nanjing, China; b Branch of National Clinical Research Center for Laboratory Medicine, Nanjing, China; c Department of Pathology, the First Affiliated Hospital of Nanjing Medical University, Nanjing, China.

**Keywords:** case report, immunotherapy, laboratory examinations, mucosa-associated lymphoid tissue lymphoma, pulmonary

## Abstract

**Rationale:**

Primary pulmonary mucosa-associated lymphoid tissue lymphoma (MALToma) is a rare subtype of non-Hodgkin lymphoma with a relatively low incidence rate clinically. Atypical clinical symptoms and nonspecific chest computed tomography features of the disease make it difficult to determine and treatment is delayed. We discuss the diagnosis and treatment of a patient with primary pulmonary MALToma to raise clinicians’ awareness of this condition.

**Patient concerns:**

A 66-year-old male patient with a medical history of tuberculosis has been experiencing progressive exacerbation of respiratory symptoms and nonresponsive treatment without an unclear diagnosis for 5 years. He was transferred to our hospital because a nonspecific soft tissue mass in the right upper lobe of the lung was found on his chest computed tomography. Laboratory results with serum immunofixation electrophoresis showed polyclonal immunoglobulin (Ig) G, IgM, IgA, and λ-light chain on admission.

**Diagnosis:**

Pathological examination and immunohistochemical staining of lung biopsy revealed a definitive diagnosis of pulmonary MALToma with stage IV.

**Interventions and outcomes:**

The patient received immunotherapy with anti-CD20 monoclonal antibody (rituximab), and showed significant clinical improvement at the 6-month follow-up.

**Conclusions and lessons:**

Diagnosis of primary pulmonary MALToma mainly relies on histopathological examination, and comprehensive laboratory examinations are also necessary. Clinicians should combine laboratory tests (such as immunofixation electrophoresis in our case) to assist in medical diagnosis in cases of atypical clinical manifestations and imaging characteristics. Immunotherapy appears to be the main treatment protocol for advanced patients.

## 1. Introduction

The concept of mucosa-associated lymphoid tissue lymphoma (MALToma) was first proposed in 1983 and has been described as a distinctive type of indolent B-cell lymphoma based on histopathological features.^[1]^ MALToma is frequently observed in the gastrointestinal tract (around 50%), but can actually involve any mucosa and occur at various sites, including the lungs (with a proportion of 15%), ocular appendages and salivary glands.^[2]^ Pulmonary MALToma is a relatively rare disease that accounts for 0.5% to 1% of primary lung neoplasms and is defined as malignant clonal proliferation of the pulmonary parenchyma or bronchial lymphoid tissue.^[3]^ Herein, we report the clinical characteristics, diagnosis process and therapeutic schedule of a rare case of primary pulmonary MALToma.

## 2. Case presentation

A 66-year-old male patient with a medical history of tuberculosis developed cough and expectoration without obvious inducement, dyspnea after activity, dizziness and tinnitus in a static state in 2014. The above symptoms worsened in 2017 and were accompanied by a large amount of white phlegm, shortness of breath at rest, night sweats, emaciation and fatigue. He was subsequently diagnosed with tuberculosis, but showed no improvement after symptomatic treatment. Chest computed tomography performed at a local hospital in 2020 revealed pneumonic lesions in both lungs, mediastinal lymph node enlargement, bilateral pleural thickening and bronchodilation changes. Meanwhile, a soft tissue mass was observed in the upper right lung lobe. However, these imaging features were ambiguous. The patient was transferred to the respiratory department of our hospital for a complete diagnostic workup.

**Figure 1.**
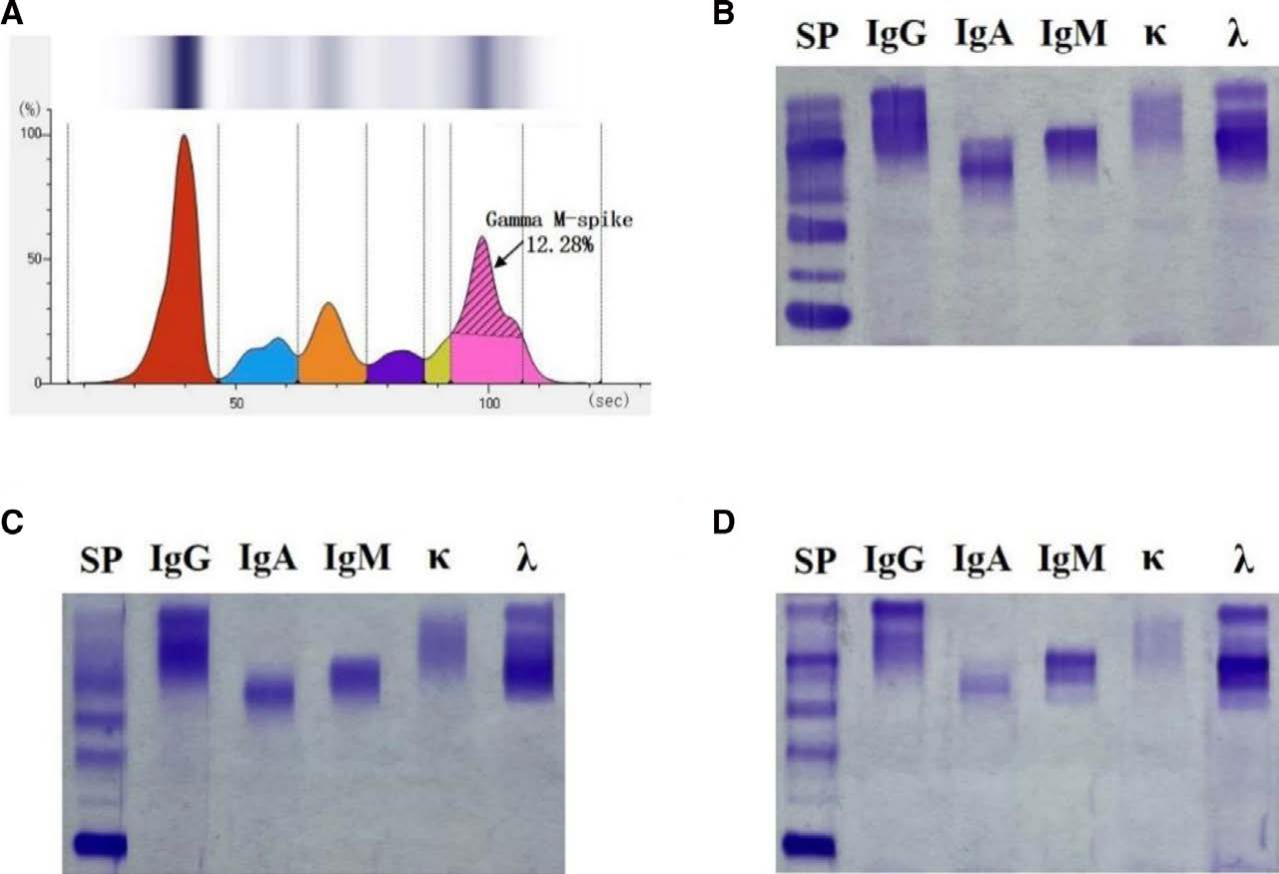
Serum protein electrophoresis and immunofixation electrophoresis. (A) Serum protein electrophoresis result of the patient upon admission; the areas with different colors from left to right are albumin, α1-globulin, α2-globulin, β1-globulin, β2-globulin, and γ-globulin regions; the percentage of albumin was used as a reference; (B) immunofixation electrophoresis of serum at admission with each lane was labeled with corresponding antiserum; (C) using 5% β-ME to process residual serum sample in (B) and repeated immunofixation electrophoresis detection; (D) serum immunofixation electrophoresis of the patient at the 6-month follow-up.

Routine color Doppler ultrasound examination revealed a large amount of pleural effusion on the right side, with a maximum anteroposterior diameter of 160 mm, which might indicate inflammation in the right lung. The main laboratory examination results on admission are presented in Table 1. It suggested inflammation (white blood cell count of 14.3 × 10^9^/L and erythrocyte sedimentation rate of 75 mm/h) and mild anemia (hemoglobin of 92 g/L) of the patient, and he was accompanied by malnutrition (serum albumin of 26.7 g/L) and elevated serum lactate dehydrogenase concentration (347 U/L) simultaneously. Notably, his serum immunoglobulin (Ig) M and λ-light chain, measured by immunonephelometry assay, increased to 13.4 g/L and 3.58 g/L, respectively. In addition to pulmonary diseases such as tuberculosis and lung cancer, hematological diseases have begun to be noticed by clinicians. After consultation with the clinical chemistry laboratory, clinicians ordered serum protein electrophoresis and immunofixation electrophoresis (IFE) detections. Serum protein electrophoresis revealed a conspicuous spike, viz. M-spike, suggesting a high monoclonal Ig level in the serum of the patient (Fig. 1A, arrows). Contrary to the results detected by INA, IFE displayed clearly visible stripes of IgG, IgM, IgA and λ-light chain (Fig. 1B). We had doubts about this inconsistency and repeated the IFE examination following the depolymerization of serum with 5% β-mercaptoethanol (β-ME) for 30 minutes. The procedure was performed to eliminate the aggregative effect of IgM itself or in combination with its surrounding proteins, and the conclusion remained the same (Fig. 1C). The above-mentioned phenomenon showed there were large amounts of abnormal Igs in the patient’s circulating blood, which more firmly strengthened the conjecture of clinicians. Therefore, the patient was referred to hematology department.

**Table 1:** Main laboratory results on admission.

Name	Analyte	Result	Reference interval
Blood routine examination	WBC, ×10^9^/L	14.3	3.5–9.5
	N, ×10^9^/L	10.1	1.8–6.3
	RBC, ×10^12^/L	3.98	4.0–5.5
	Hb, g/dL	92	120–160
	HCT, %	29.2	40.0–50.0
	MCV, fL	73.3	80.0–100.0
	RDW-CV, %	15.1	9.0–16.0
	PLT, ×10^9^/L	212	100–300
	ESR, mm/h	75	<38
Biochemical examination	TP, g/L	64.9	65.0–85.0
	ALB, g/L	26.7	40.0–55.0
	ALT, U/L	29.7	9.0–50.0
	AST, U/L	53.2	8.0–40.0
	ALP, U/L	122.4	45–125
	LDH, U/L	347	120–250
	*β*2-MG, mg/L	5.2	0.9–3.1
	Na, mm/L	127.9	137.0-147.0
	Cl, mm/L	94.0	99.0–110.0
	Ca, mm/L	2.01	2.11–2.52
SPE	SPE	**M-spike detected 12.28%**	No M-spike
INA	Serum IgG, g/L	7.75	7.0–16.0
	Serum IgM, g/L	13.4	0.4–2.3
	Serum IgA, g/L	2.52	0.7–4.0
	Serum free κ, g/L	1.07	1.7–3.7
	Serum free λ, g/L	3.58	0.9–2.1
	Serum free κ/λ ratio	0.3	1.35–2.65
IFE	Serum IFE	**IgG, IgM, IgA and λ-light chain**	No monoclonal
Pathogen detection	HBV, IU/mL	3.62 × 10^3^	<100
	Acid-fast bacillus	No acid-fast bacillus	No acid-fast bacillus
	T-SPOT	Negative	Negative

ALB = albumin, ALP = alkaline phosphate, ALT = alanine aminotransferase, AST = aspartate amino transferase, ESR = erythrocyte sedimentation rate, Hb = hemoglobin, HBV = hepatitis B virus, HCT = hematocrit, IFE = immunofixation electrophoresis, LDH = lactate dehydrogenase, MCV = mean corpuscular volume, N = neutrophils count, PLT = platelet count, RBC = red blood cell count, RDW-CV = red blood cell distribution width-coefficient of variation, SPE = serum protein electrophoresis, TP = total protein, T-SPOT = T cell spot test for tuberculosis infection, WBC = white blood cell count, β2-MG = β2 microglobulin.

**Figure 2.**
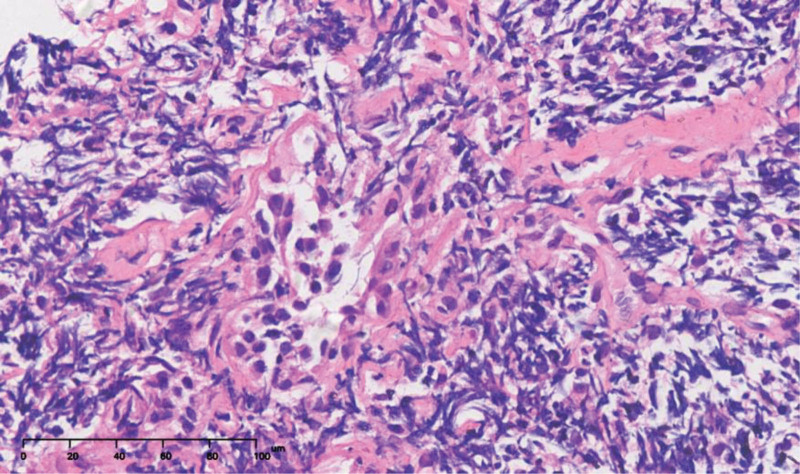
Pathological section of the right lung mass (HE staining, magnification × 200). Lung tissue showed scattered adenoid structure with peripherally diffuse infiltration of lymphocytes B of different sizes.

**Figure 3.**
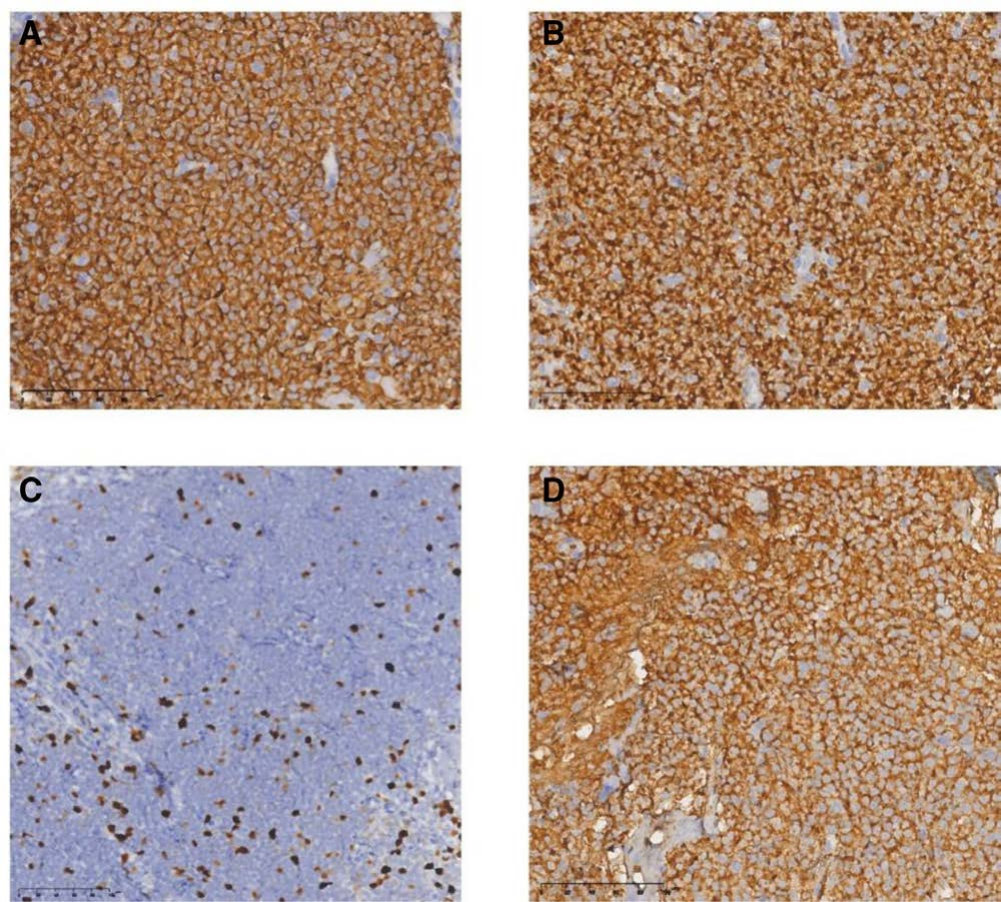
Immunohistochemical staining characteristics of lung tissue. Tumor cells were positive for CD20 (A), Bcl-2 (B), and λ light chain (D) expression (magnification × 100, respectively); 15% to 20% of Ki-67 reaction is positive (C, magnification × 50). Numerous lymphocytes B can be seen in the tissue.

**Figure 4.**
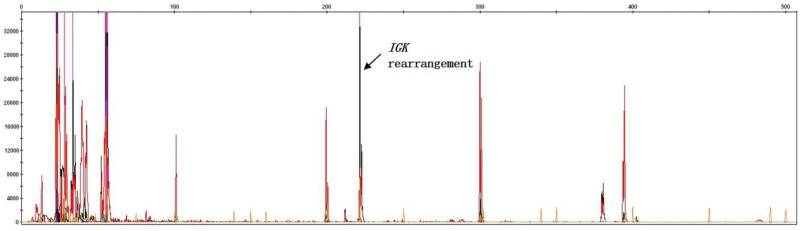
*IGK* rearrangement detection of bone marrow. The arrow indicated a positive *IGK* rearrangement, while that of normal individuals is negative.

HE staining of the lung tissue biopsy showed a scattered adenoid structure with peripherally diffuse infiltration of B lymphocytes of different sizes (Fig. 2). Immunohistochemical analysis was positive for cluster of differentiation (CD) 20, CD21, CD79a, Bcl-2, Bcl-6, Ki-67 (15–20%) and λ-light chain expression (Fig. 3A–D), which are characteristic markers of MALToma as previously reported,^[4]^ but negative for CD10, CD43, cyclin D, keratin AE1/AE3, epithelial membrane antigen (EMA), thyroid transcription factor 1 (TTF-1) and κ-light chain expression. Additionally, bone marrow gene molecular testing showed that *T-cell receptor (TCR)β, TCRγ* and *TCRδ* rearrangements were negative, whereas that of *immunoglobulin light chain kappa (IGK*) was positive (Fig. 4), revealing the abnormal lymphocytes proliferation and was reported could provide early guidance for lymphoma diagnosis.^[5]^

The pathologist and clinician confirmed the suspected diagnosis of stage IV pulmonary MALToma, along with chronic hepatitis B virus (HBV) infection according to the location of the lesion and a series of inspection results, including pathological examination and laboratory tests. The patient agreed to receive immunotherapy with anti-CD20 monoclonal antibody (rituximab) and antiviral (entecavir and valacyclovir) therapy, and followed blood indicators monitor weekly. After 6-month medication on time continuously, the clinical symptoms of the patient were relieved, and the serum levels of IgA (Fig. 1D) and HBV decreased to be undetectable.

## 3. Discussion

Pulmonary MALToma is the most common type (approximately 70%–90%) of primary lung lymphoma (PPL), which is a rare clonal abnormal hyperplasia disease involving one or both lungs with no evidence of extrapulmonary involvement within 3 months after diagnosis.^[4,6]^ In addition, the probability of PPL involving the right side is higher than that involving the left side.^[7]^ The disease is recognized as a unique extranodal marginal zone lymphoma according to the World Health Organization (WHO) classification.^[8]^ Pulmonary MALToma has recently been reported to have a median age of approximately 60 years at diagnosis^[9]^ and an equal incidence rate in males and females.^[10]^

The etiology that could play a role in the pathogenesis of pulmonary MALToma is yet to be completely elucidated, but it is currently widely accepted that immune cross-reactions caused by chronic inflammation (including smoking, autoimmune stimuli, and infectious agents such as bacteria and viruses) participate in this process.^[11]^ The patient in our case had a history of tuberculosis; although it had been cured, the lungs were continuously exposed to the stimulation of *Mycobacterium tuberculosis* antigens before. The complication of HBV infection indicated the presence of chronic immune stimulation of antigens internally. These chronic stimuli induce pulmonary inflammation and simultaneously become risk factors for pulmonary MALToma, which is consistent with the pathogenesis of the disease. However, there is no report on how HBV and *M tuberculosis* affect the progression of pulmonary MALToma concretely, which is worth further exploration.

The onset of pulmonary MALToma is latent and slow, patients normally lack typical or specific clinical symptoms in the early stages and generally exhibit nonspecific recurrent respiratory infections, such as cough, dyspnea, fatigue, and chest pain.^[12]^ However, nearly half of the patients are asymptomatic at their first visit,^[13]^ which easily causes negligence. Weight loss, hemoptysis and fever may be considered as possible transformations to an aggressive form.^[14]^ According to the patient’s description, he had developed night sweats, emaciation and fatigue in 2017; coupled with the low specificity of chest computed tomography features and medical history, it was easily misdiagnosed with pulmonary tuberculosis. The final treatment outcome was undoubtedly failed and worsened disease progression. Histopathological examinations, immunohistochemistry, molecular markers and gene rearrangement studies after surgery are considered effective methods for the confirmation of pulmonary MALToma.^[15]^ The IFE results in this case seem to serve as a reminder. A study revealed that abnormally proliferative monoclonal Igs in serum were helpful for refining the risk assessment of MALToma patients^[16]^ and presented a better ability to predict progression-free survival than International Prognostic Index scores.^[17]^ IFE has been reported to be of great significance in correctly evaluating circulating paraproteins and identifying their immune subtypes in MALToma patients owing to its high detection sensitivity.^[18]^ Therefore, in response to low-level immunoglobulin paraproteinemia, IFE avoids the underestimation of MALToma.

Due to the small number of MALToma patients worldwide, standard therapeutic guidelines for the disease have not been established. The main principle is to combine various variables related to the patient, the disease and the therapeutic schedule. Based on this principle, clinicians choose one or more approaches, including surgery, chemotherapy, radiotherapy (RT), immunotherapy and watch-and-wait (W&W).^[19]^ Thoracoscopic surgery is the best option for localized resectable lesions of pulmonary MALToma. For patients with non-resectable lesions or contraindications, chemotherapy, RT or immunotherapy can be used. It has been proven that rituximab can alleviate disease progression in 70% of patients with pulmonary MALToma, whereas RT can effectively control localized lesions in patients with stages I to II.^[11,20]^ In addition, W&W is suitable for asymptomatic patients because of the indolent, aggressive and spontaneous disease characteristics.^[11]^ Moreover, antibiotic or targeted therapy has also been shown to be effective in managing pulmonary MALToma-related pathogens.^[21]^ New therapeutic strategies have emerged in which ibrutinib is validated to be effective and safe in patients with relapses or refractory to rituximab, and PI3K inhibitors (copanlisib and umbrasilib) show excellent activity.^[11]^ Pulmonary MALToma exhibits a favorable prognosis with a 5-year survival rate of over 80% after correct diagnosis and treatment.^[22]^ It is recommended that reexaminations be conducted every 6 months for the first 5 years and annually thereafter.^[19]^

## 4. Conclusion

Pulmonary MALToma is a rare form of PPL, and its clinical symptoms and imaging features are diverse and atypical, posing great challenges for clinicians. Although the best diagnostic method for this disease is lung tissue biopsy, comprehensive laboratory examinations are necessary for persistent and incurable respiratory diseases. There are no unified therapeutic guidelines, which require comprehensive consideration. Patients with pulmonary MALToma can achieve a long survival time with timely and personalized treatment. More effective diagnostic methods and therapeutic schedules for pulmonary MALToma may be key points that should be focused on in future studies.

## Acknowledgments

We thank Dr Bubin Wang from Southeast University for his knowledge sharing and comments on modifications.

## Author contributions

**Conceptualization:** Chengjing Yan.

**Investigation:** Yanfei Lu.

**Methodology:** Min Gu.

**Resources:** Guoqiang Ping.

**Visualization:** Min Gu, Dongze Ji.

**Writing – original draft:** Min Gu, Dongze Ji, Yanfei Lu.

**Writing – review & editing:** Chengjing Yan.
